# The Regulatory Role of Nuclear Factor Kappa B in the Heart of Hereditary Hypertriglyceridemic Rat

**DOI:** 10.1155/2016/9814038

**Published:** 2016-04-11

**Authors:** Stanislava Vranková, Andrej Barta, Jana Klimentová, Ima Dovinová, Silvia Líšková, Zdenka Dobešová, Oľga Pecháňová, Jaroslav Kuneš, Josef Zicha

**Affiliations:** ^1^Institute of Normal and Pathological Physiology and Centre of Excellence for Regulatory Role of Nitric Oxide in Civilization Diseases, Slovak Academy of Sciences, 813 71 Bratislava, Slovakia; ^2^Institute of Physiology, Academy of Sciences of the Czech Republic, 142 20 Prague 4, Czech Republic; ^3^Centre of Cardiovascular Research, 142 20 Prague 4, Czech Republic

## Abstract

Activation of nuclear factor-*κ*B (NF-*κ*B) by increased production of reactive oxygen species (ROS) might induce transcription and expression of different antioxidant enzymes and also of nitric oxide synthase (NOS) isoforms. Thus, we aimed at studying the effect of NF-*κ*B inhibition, caused by JSH-23 (4-methyl-*N* ^1^-(3-phenyl-propyl)-benzene-1,2-diamine) injection, on ROS and NO generation in hereditary hypertriglyceridemic (HTG) rats. 12-week-old, male Wistar and HTG rats were treated with JSH-23 (bolus, 10 *μ*mol, i.v.). After one week, blood pressure (BP), superoxide dismutase (SOD) activity, SOD1, endothelial NOS (eNOS), and NF-*κ*B (p65) protein expressions were higher in the heart of HTG rats compared to control rats. On the other hand, NOS activity was decreased. In HTG rats, JSH-23 treatment increased BP and heart conjugated dienes (CD) concentration (measured as the marker of tissue oxidative damage). Concomitantly, SOD activity together with SOD1 expression was decreased, while NOS activity and eNOS protein expression were increased significantly. In conclusion, NF-*κ*B inhibition in HTG rats led to decreased ROS degradation by SOD followed by increased oxidative damage in the heart and BP elevation. In these conditions, increased NO generation may represent rather a counterregulatory mechanism activated by ROS. Nevertheless, this mechanism was not sufficient enough to compensate BP increase in HTG rats.

## 1. Introduction

Increase in cell production of reactive oxygen species (ROS) leads to activation of intracellular signaling pathways, which in turn induce transcriptional changes that enable a cell to activate expression of a number of genes encoding antioxidant proteins, DNA repair proteins, stress-regulated chaperones, and antiapoptotic proteins. These genes are generally regulated by transcription factors whose structure, subcellular localization, or affinity for DNA is directly or indirectly regulated by the level of oxidative stress [1]. In such a way, ROS may serve as messenger molecules to activate adaptive responses, such as redox-sensitive nuclear factor kappa B (NF-*κ*B) signaling, which enhance gene expression of antioxidant enzymes in oxidatively stressed tissue [2, 3]. In addition, NF-*κ*B may participate in regulation of nitric oxide synthase (NOS) isoforms expression including eNOS [4, 5].

The transcription factor NF-*κ*B has been shown to be cardioprotective after permanent coronary occlusion and late ischemic preconditioning. However, cell injurious effect of this factor after ischemia/reperfusion was shown in the heart as well. Tranter et al. identified 16 NF-*κ*B dependent cardioprotective genes that might contribute to understanding the mechanism of NF-*κ*B-induced myocardial salvage after permanent coronary occlusion [6].

NF-*κ*B belongs to the Rel family of transcriptional activator proteins and it exerts a variety of actions [7]. Sen et al. have found that NF-*κ*B responds directly to oxidative stress and its activation is controlled by the cell glutathione disulphide/glutathione (GSSG/GSH) ratio [8]. On the other hand,* in vitro* inhibition of the transcriptional activity of NF-*κ*B may lead to accumulation of reactive oxygen species following oxidative damage [9]. The contradictory findings on the role of NF-*κ*B signaling reflect the diversity of cellular processes on molecular level and should be taken into account in different therapeutic settings.

It is evident that the model of nonobese hereditary hypertriglyceridemic (HTG) rats selected from the Wistar strain [10] represents a suitable model for the study of metabolic disturbances in relation to blood pressure as well as in the search for genetic determinants of these abnormalities [11]. Hereditary HTG rats exhibit insulin resistance, hyperinsulinemia, disturbances in glucose metabolism, hypertension, and different signs of oxidative stress, for example, increased lipoprotein oxidability and lipid peroxidation [12]. According to our knowledge, the role of NF-*κ*B signaling in response to increased oxidative damage in HTG rats was not studied as yet.

Shin et al. [13] showed that aromatic diamine, 4-methyl-*N* ^1^-(3-phenyl-propyl)-benzene-1,2-diamine (JSH-23), had an inhibitory effect on NF-*κ*B transcriptional activity in lipopolysaccharide- (LPS-) stimulated macrophages RAW 264.7. JSH-23 had inhibitory effects, in parallel, on LPS-induced DNA binding activity and nuclear translocation of NF-*κ*B p65. However, the compound JSH-23 did not influence LPS-induced inhibitory kappa B alpha protein (I*κ*B*α*) degradation. These results indicate that the JSH-23 could inhibit nuclear translocation of NF-*κ*B p65 without affecting I*κ*B*α* degradation, which is a very rare mode of action, lending JSH-23 a specific character of NF-*κ*B inhibition.

In this study we investigated the effect of NF-*κ*B inhibition (caused by JSH-23 injection) on heart reactive oxygen species level, superoxide dismutase and nitric oxide synthase activities, and blood pressure regulation in hereditary hypertriglyceridemic rats.

## 2. Material and Methods

### 2.1. Animals and Treatment

Male 12-week-old normotensive Wistar rats and Prague hereditary hypertriglyceridemic (HTG) rats (bred in the Institute of Physiology AS CR, Prague) were used in this study. All animals were kept under standard laboratory conditions (12 h light, 12 h darkness, 23 ± 1°C, pelleted ST-1 diet, drinking* ad libitum*). All procedures and experimental protocols were approved by the Animal Care Ethical Committee of the Institute of Physiology AS CR in Prague and conformed to the European Convention on Animal Protection and Guidelines on Research Animal Use. Adult 12-week-old Wistar (*n* = 18) and HTG (*n* = 18) rats were included in the study. Nine Wistar rats and 9 HTG rats were taken as controls, whereas the remaining rats (9 Wistar and 9 HTG) were injected with JSH-23 (bolus, 10 *μ*mol, i.v.).

At the end of the experiment, one week after JSH-23 injection, blood pressure was measured by a direct puncture of the carotid artery under light ether anesthesia. Heart was dissected and left ventricle (LV) was taken for determination of biochemical parameters.

### 2.2. Biochemical Parameters

The concentration of conjugated dienes (CD) was measured in lipid extracts of heart homogenates [14]. After chloroform evaporation under inert atmosphere and addition of cyclohexane, conjugated diene concentrations were determined spectrophotometrically (*λ* = 233 nm, GBC 911A, Bio-Rad Laboratories).

Reduced glutathione (GSH) level was determined according to Ellman [15]. Samples of LV were homogenized in 1 mL of ice-cold 3% sulphosalicylic acid and, after centrifugation at 3.000 ×g for 5 min, GSH concentration was determined spectrophotometrically in the acid-soluble fractions (*λ* = 412 nm, GBC 911A, Bio-Rad Laboratories).

Total NO synthase (NOS) activity was determined in crude LV homogenates by measuring L-[^3^H]citrulline formation from L-[^3^H]arginine (Amersham, UK) as previously described by Bredt and Snyder [16] with minor modifications [17, 18].

Superoxide dismutase (SOD) activity was analyzed in LV homogenates spectrophotometrically using the SOD assay kit (Fluka, Switzerland). The absorbance was measured at 450 nm using a microplate reader (Thermo Scientific Multiscan FC, Finland). SOD activity was expressed in U/mg of protein in the tissues.

For Western blot analysis, samples of the LV were used and probed with polyclonal rabbit anti-eNOS (Santa Cruz Biotechnology, USA), anti-SOD1 (Santa Cruz Biotechnology, USA), anti-NF*κ*B (p65) (BioLegend, USA), and anti-*β*-actin (Santa Cruz Biotechnology, USA) antibodies.

### 2.3. Statistical Analysis

Results are expressed as means ± SEM. One-way ANOVA and Bonferroni test were used for statistical analysis. *p* < 0.05 value was considered statistically significant.

## 3. Results

### 3.1. Biometric Parameters

At the end of experiment, mean blood pressure of control HTG rats was significantly increased (by 40%) in comparison with control Wistar rats. After JSH-23 treatment, blood pressure was increased significantly only in HTG rats compared with age-matched untreated rats (Table 1).

HTG rats had lower body weight and heart weight in comparison with normotensive Wistar rats. In HTG rats, HW/BW ratio was significantly higher than in Wistar rats. JSH-23 administration did not affect body weight, heart weight, and relative heart weight of Wistar as well as HTG rats (Table 1).

### 3.2. Biochemical Parameters

#### 3.2.1. NF-*κ*B (p65) Expression and CD and GSH Concentration

**Figure 1:**
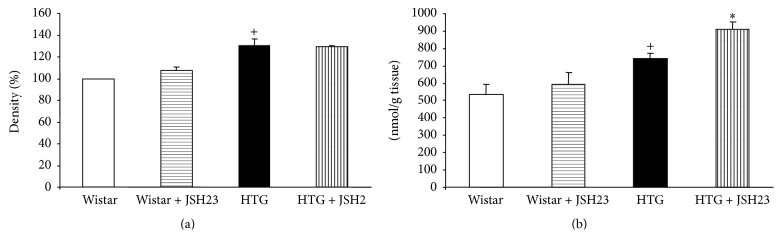
NF-*κ*B protein expression (a) and conjugated diene concentration (b) in the heart. HTG, hypertriglyceridemic; JSH-23, 4-methyl-*N* ^1^-(3-phenyl-propyl)-benzene-1,2-diamine. Data are means ± SEM (*n* = 9). ^+^ *p* < 0.05 as compared to Wistar rats; ^*∗*^ *p* < 0.05 as compared to controls.

The protein expression of NF-*κ*B (subunit p65) was significantly higher in HTG rats than in Wistar rats. JSH-23 treatment had no effect on NF-*κ*B protein expression in HTG as well as Wistar rats (Figure 1(a)).

The levels of conjugated dienes were increased significantly in HTG rats as compared to Wistar rats. JSH-23 increased concentration of conjugated dienes only in HTG rats (Figure 1(b)).

No significant changes in GSH levels were seen in HTG rats versus Wistar rats or in HTG rats and Wistar rats treated with JSH-23 (data not shown).

#### 3.2.2. NOS Activity and eNOS Expression

**Figure 2:**
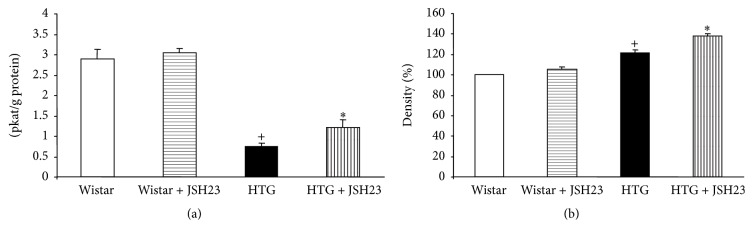
Total NOS activity (a) and endothelial NOS protein expression (b) in the heart. HTG, hypertriglyceridemic; JSH-23, 4-methyl-*N* ^1^-(3-phenyl-propyl)-benzene-1,2-diamine. Data are means ± SEM (*n* = 9). ^+^ *p* < 0.05 as compared to Wistar rats; ^*∗*^ *p* < 0.05 as compared to controls.

Total NOS activity was significantly decreased in HTG rats in comparison with Wistar rats. JSH-23 treatment increased NOS activity in HTG rats and there were no significant changes in Wistar rats (Figure 2(a)).

Endothelial NO synthase (eNOS) protein expression was increased significantly in HTG rats. JSH-23 administration did not affect eNOS expression in control Wistar rats, but it was significantly increased in HTG rats (Figure 2(b)).

#### 3.2.3. SOD Activity and SOD1 Expression

**Figure 3:**
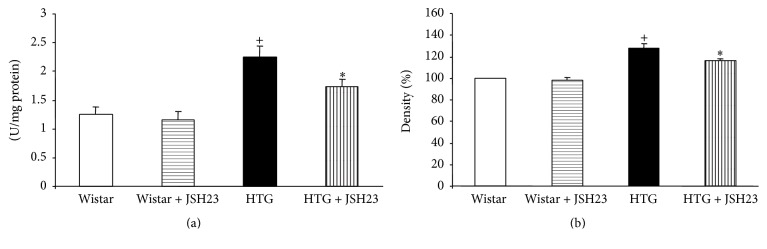
Superoxide dismutase activity (a) and SOD1 protein expression (b) in the heart. HTG, hypertriglyceridemic; JSH-23, 4-methyl-*N* ^1^-(3-phenyl-propyl)-benzene-1,2-diamine. Data are means ± SEM (*n* = 9). ^+^ *p* < 0.05 as compared to Wistar rats; ^*∗*^ *p* < 0.05 as compared to controls.

SOD activity was significantly increased in HTG rats in comparison with Wistar rats. However, SOD activity decreased significantly in HTG rats treated with JSH-23 as compared to untreated HTG rats. JSH-23 treatment had no significant effect on SOD activity in Wistar rats (Figure 3(a)).

Protein expression of SOD1 was increased in HTG rats (versus Wistar rats). JSH-23 attenuated the expression of SOD1 only in HTG rats (Figure 3(b)).

## 4. Discussion

In the present study, the aromatic diamine JSH-23 compound was used for the first time to inhibit NF-*κ*B transcriptional activity in the model of hereditary hypertriglyceridemic rats. In this model we detected increased BP and heart hypertrophy together with increased CD concentration, SOD activity, and SOD1, eNOS, and NF-*κ*B (p65) protein expressions. On the other hand, NOS activity was decreased significantly. NF-*κ*B inhibition led to additional increase in blood pressure and CD concentration, decrease in SOD1 expression and SOD activity, and, interestingly, increase in eNOS expression followed by elevated NOS activity. These results suggested rather regulatory than pathological role of NF-*κ*B in HTG rats.

Heart hypertrophy observed in HTG rats in our experiments is in accordance with previous studies, in which increase in blood pressure about 20–40 mmHg represents a hemodynamic overload that induced left ventricular hypertrophy [19]. The serious role of superoxides in blood pressure maintenance of moderately hypertensive HTG rats was demonstrated by Kuneš et al. using acute i.v. tempol administration in conscious animals [20]. Zicha et al. reported that the concentration of conjugated dienes, a marker of oxidative membrane damage, was significantly increased in the kidney of both 3- and 7-month-old HTG rats in comparison with Wistar rats. On the other hand, baseline GSH/GSSG ratio, as a marker of redox control, was significantly higher in 12-week-old HTG rats than in age-matched Wistar rats [11]. This increase was probably caused by activation of antioxidant mechanisms in hypertriglyceridemic animals, in which higher production of reactive oxygen species was documented [20, 21]. Oxidative stress manifestation was further enhanced by high-sucrose diet, as demonstrated by increased TBARS and conjugated diene concentration, decreased GSH levels, and decreased glutathione peroxidase activity in blood and liver of this respective animal model [22].

Increased level of reactive oxygen species may represent an initial step in the signal cascade of NF-*κ*B activation [2, 5]. Consequently, transcription factor NF-*κ*B enhances gene expression of antioxidant enzymes in oxidatively stressed tissue [2]. In our experiment, NF-*κ*B inhibition by JSH-23 showed decreased SOD1 expression and SOD activity together with increased CD concentration in the heart of HTG rats. On the other hand, GSH level was not affected by this treatment. Previously we have reported that chronic NF-*κ*B inhibition with lactacystin also increased CD concentration in the heart of *N* ^G^-nitro-L-arginine methyl ester- (L-NAME-) treated rats [23]. Similarly, other authors showed that lactacystin treatment significantly increased oxidative protein damage (measured as the level of protein carbonyls), lipid peroxidation, and concentration of 3-nitrotyrosine in cell culture [24]. Higher levels of lactacystin increased the concentrations of 8-hydroxyguanine (a biomarker of oxidative DNA damage) and decreased GSH levels. Lactacystin treatment also decreased significantly activity of superoxide dismutase 1 and 2 [24]. Previously, Chen et al. documented accumulation of reactive oxygen species after inhibition of the NF-*κ*B transcriptional activity also [9].

It has been suggested that intracellular ROS overproduction may represent one of the causes leading to increased blood pressure in both experimental models and human hypertension [25–27]. Considering that the reactions between NO and superoxide anion are most likely a major cause of impaired endothelium dependent vasorelaxation in hypertension [28]. As mentioned above, a higher production of reactive oxygen species was also documented in HTG rats. In our study, NF-*κ*B inhibition by JSH-23 led to a slight but significant blood pressure increase in HTG rats in comparison with untreated animals. We hypothesize that elevated production of ROS after NF-*κ*B inhibition might play an important role in blood pressure increase observed in this experimental model. Similarly, our recent results indicated that chronic NF-*κ*B inhibition with lactacystin increased blood pressure in L-NAME-treated rats [23]. Moreover, in this study, JSH-23 treatment increased total NOS activity in the heart of HTG rats. Similarly, lactacystin treatment led to increased production of nitric oxide, measured as levels of NO_2_^−^ plus NO_3_^−^ in cells [24]. Finally, we observed increased endothelial NO synthase expression in the heart of HTG rats treated with JSH-23. These changes could be explained as a compensatory mechanism activated due to the ROS and blood pressure increase.

While excessive amounts of ROS can be harmful within the cells [29], their increase to the regulatory level may trigger different signal transduction pathways [30, 31]. Dröge et al. [32] demonstrated that ROS elevated intracellular GSSG level and thereby acted indirectly on the signal cascade of NF-*κ*B activation, because NF-*κ*B activation requires an altered level of GSSG. Activated NF-*κ*B then enhances gene expression of antioxidant enzymes [2]. In our study, the inhibition of NF-*κ*B protein by JSH-23 caused inactivation of NF-*κ*B signaling, blocking presumably also the synthesis of antioxidants. Indeed, we detected decreased superoxide dismutase activity and SOD1 expression in the heart of HTG rats treated with JSH-23. Our present results confirm the role of NF-*κ*B signaling in gene expression of antioxidant enzymes in oxidatively stressed tissue. Cho et al. showed several possible mechanisms responsible for cellular ROS accumulation induced by NF-*κ*B inhibition. The most important ones involve impairment of the activities of antioxidant enzymes and glutathione depletion by NF-*κ*B inhibition [33]. Moreover, the production of antioxidant enzymes, such as ferritin heavy chain, manganese-dependent superoxide dismutase, and metallothionein, was found to exhibit NF-*κ*B-dependent manner [34]. A study by Cho et al. also reported that NF-*κ*B inhibition increased superoxide anion level and decreased GSH level in isolated human CD4^+^ T cells [33]. Reduced GSH is a major intracellular antioxidant and NF-*κ*B is the most important transcription factor, which induces the gene for glutamylcysteine synthetase, the rate-limiting enzyme for GSH synthesis [35]. NF-*κ*B inhibitors, including pyrrolidine dithiocarbamate [36], parthenolide [37], gliotoxin [38], and proteosome inhibitor [24], were reported to cause GSH depletion. However, our present study did not confirm the decrease of GSH level after JSH-23 treatment. The unchanged GSH level along with increased eNOS protein expression after JSH-23 treatment in the heart may represent a compensatory mechanism activated due to ROS accumulation and blood pressure increase in HTG rats.

In conclusion, our data show that NF-*κ*B inhibition by JSH-23 leads to further increase of oxidative damage followed by increased blood pressure in the model of hereditary hyperglyceridemic rats. Under these conditions, increased NO production represents rather counterbalancing mechanism activated by blood pressure increase in this respective model. Thus, NF-*κ*B inhibition under increased ROS level may not always have a beneficial effect in the heart.

## Figures and Tables

**Table 1:** Body weight, heart weight, relative heart weight, and mean arterial pressure of Wistar rats. Wistar rats treated with JSH-23, hereditary hypertriglyceridemic (HTG) rats, and hereditary hypertriglyceridemic rats treated with JSH-23.

	BW (g)	HW (mg)	HW/BW mg/100 g	MAP (mmHg)
Wistar	435 ± 4	1074 ± 24	246 ± 4	95 ± 2
Wistar + JSH-23	426 ± 5	1039 ± 28	244 ± 4	98 ± 4
HTG	328 ± 6^+^	890 ± 32^+^	271 ± 7^+^	133 ± 4^+^
HTG + JSH-23	344 ± 10	965 ± 23	280 ± 3	144 ± 3^*∗*^

BW, body weight; HW, heart weight; HTG, hypertriglyceridemic; HW/BW, heart weight to body weight ratio; JSH-23, 4-methyl-*N* ^1^-(3-phenyl-propyl)-benzene-1,2-diamine; MAP, mean arterial pressure. Data are means ± SEM; significant differences ^+^ *p* < 0.05 compared with Wistar rats; ^*∗*^ *p* < 0.05 compared with control rats.

## Abbreviations

BP: Blood pressure
CD: Conjugated dienes
GSH: Reduced glutathione
GSSG: Glutathione disulphide
HTG: Hypertriglyceridemic
JSH-23: 4-Methyl-*N* ^1^-(3-phenyl-propyl)-benzene-1,2-diamine
L-NAME: *N* ^G^-Nitro-L-arginine methyl ester
NF-*κ*B: Nuclear factor *κ*B
NO: Nitric oxide
NOS: NO synthase
eNOS: Endothelial NOS
ROS: Reactive oxygen species
SOD: Superoxide dismutase
TBARS: Thiobarbituric Acid Reactive Substances.

## References

[1] Liu H., Colavitti R., Rovira I. I., Finkel T. (2005). Redox-dependent transcriptional regulation. *Circulation Research*.

[2] Bar-Shai M., Carmeli E., Ljubuncic P., Reznick A. Z. (2008). Exercise and immobilization in aging animals: the involvement of oxidative stress and NF-*κ*B activation. *Free Radical Biology and Medicine*.

[3] Kwak J.-H., Jung J.-K., Lee H. (2011). Nuclear factor-kappa B inhibitors; a patent review (2006–2010). *Expert Opinion on Therapeutic Patents*.

[4] Grumbach I. M., Chen W., Mertens S. A., Harrison D. G. (2005). A negative feedback mechanism involving nitric oxide and nuclear factor kappa-B modulates endothelial nitric oxide synthase transcription. *Journal of Molecular and Cellular Cardiology*.

[5] Morgan M. J., Liu Z.-G. (2011). Crosstalk of reactive oxygen species and NF-*κ*B signaling. *Cell Research*.

[6] Tranter M., Ren X., Forde T. (2010). NF-*Κ*B driven cardioprotective gene programs; Hsp70.3 and cardioprotection after late ischemic preconditioning. *Journal of Molecular and Cellular Cardiology*.

[7] Napetschnig J., Wu H. (2013). Molecular basis of NF-*κ*B signaling. *Annual Review of Biophysics*.

[8] Sen C. K., Khanna S., Reznick A. Z., Roy S., Packer L. (1997). Glutathione regulation of tumor necrosis factor-*α*-induced NF-*κ*B activation in skeletal muscle-derived L6 cells. *Biochemical and Biophysical Research Communications*.

[9] Chen F., Castranova V., Li Z., Karin M., Shi X. (2003). Inhibitor of nuclear factor kappa B kinase deficiency enhances oxidative stress and prolongs c-Jun NH2-terminal kinase activation induced by arsenic. *Cancer Research*.

[10] Vrána A., Kazdová L. (1990). The hereditary hypertriglyceridemic nonobese rat: an experimental model of human hypertriglyceridemia. *Transplantation Proceedings*.

[11] Zicha J., Pecháňová O., Čačányiová S. (2006). Hereditary hypertriglyceridemic rat: a suitable model of cardiovascular disease and metabolic syndrome?. *Physiological Research*.

[12] Kazdová L., Žák A., Vrána A. (1997). Increased lipoprotein oxidability and aortic lipid peroxidation in an experimental model of insulin resistance syndrome. *Annals of the New York Academy of Sciences*.

[13] Shin H.-M., Kim M.-H., Kim B. H. (2004). Inhibitory action of novel aromatic diamine compound on lipopolysaccharide-induced nuclear translocation of NF-*κ*B without affecting I*κ*B degradation. *FEBS Letters*.

[14] Kogure K., Watson B. D., Busto R., Abe K. (1982). Potentiation of lipid peroxides by ischemia in rat brain. *Neurochemical Research*.

[15] Ellman G. L. (1959). Tissue sulfhydryl groups. *Archives of Biochemistry and Biophysics*.

[16] Bredt D. S., Snyder S. H. (1990). Isolation of nitric oxide synthetase, a calmodulin-requiring enzyme. *Proceedings of the National Academy of Sciences of the United States of America*.

[17] Pecháňová O., Bernátová I., Pelouch V., Šimko F. (1997). Protein remodelling of the heart in NO-deficient hypertension: the effect of captopril. *Journal of Molecular and Cellular Cardiology*.

[18] Pecháňová O., Zicha J., Kojšová S., Dobešová Z., Jendeková L., Kuneš J. (2006). Effect of chronic *N*-acetylcysteine treatment on the development of spontaneous hypertension. *Clinical Science*.

[19] Simko F., Luptak I., Matuskova J. (2002). Heart remodeling in the hereditary hypertriglyceridemic rat: Effect of captopril and nitric oxide deficiency. *Annals of the New York Academy of Sciences*.

[20] Kuneš J., Dobešová Z., Zicha J. (2002). Altered balance of main vasopressor and vasodepressor systems in rats with genetic hypertension and hypertriglyceridaemia. *Clinical Science*.

[21] Žourek M., Kyselová P., Mudra J. (2008). The relationship between glycemia, insulin and oxidative stress in hereditary hypertriglyceridemic rat. *Physiological Research*.

[22] Škottová N., Kazdová L., Oliyarnyk O., Večeřa R., Sobolová L., Ulrichová J. (2004). Phenolics-rich extracts from *Silybum marianum* and *Prunella vulgaris* reduce a high-sucrose diet induced oxidative stress in hereditary hypertriglyceridemic rats. *Pharmacological Research*.

[23] Vranková S., Parohová J., Barta A., Janega P., Šimko F., Pecháňová O. (2010). Effect of nuclear factor kappa B inhibition on L-NAME-induced hypertension and cardiovascular remodelling. *Journal of Hypertension*.

[24] Lee M. H., Hyun D.-H., Jenner P., Halliwell B. (2001). Effect of proteasome inhibition on cellular oxidative damage, antioxidant defences and nitric oxide production. *Journal of Neurochemistry*.

[25] Ward N. C., Croft K. D. (2006). Hypertension and oxidative stress. *Clinical and Experimental Pharmacology and Physiology*.

[26] Kojšová S., Jendeková L., Zicha J., Kuneš J., Andriantsitohaina R., Pecháňová O. (2006). The effect of different antioxidants on nitric oxide production in hypertensive rats. *Physiological Research*.

[27] Wilcox C. S., Pearlman A. (2008). Chemistry and antihypertensive effects of tempol and other nitroxides. *Pharmacological Reviews*.

[28] Skibska B., Goraca A. (2015). The protective effect of lipoic acid on selected cardiovascular diseases caused by age-related oxidative stress. *Oxidative Medicine and Cellular Longevity*.

[29] Shaw P. X., Werstuck G., Chen Y. (2014). Oxidative stress and aging diseases. *Oxidative Medicine and Cellular Longevity*.

[30] Valko M., Leibfritz D., Moncol J., Cronin M. T. D., Mazur M., Telser J. (2007). Free radicals and antioxidants in normal physiological functions and human disease. *International Journal of Biochemistry and Cell Biology*.

[31] Woodward M., Croft K. D., Mori T. A. (2009). Association between both lipid and protein oxidation and the risk of fatal or non-fatal coronary heart disease in a human population. *Clinical Science*.

[32] Dröge W., Schulze-Osthoff K., Mihm S. (1994). Functions of glutathione and glutathione disulfide in immunology and immunopathology. *The FASEB Journal*.

[33] Cho M.-L., Moon Y.-M., Heo Y.-J. (2009). NF-*κ*B inhibition leads to increased synthesis and secretion of MIF in human CD4^+^ T cells. *Immunology Letters*.

[34] Sasazuki T., Okazaki T., Tada K. (2004). Genome wide analysis of TNF-inducible genes reveals that antioxidant enzymes are induced by TNF and responsible for elimination of ROS. *Molecular Immunology*.

[35] Townsend D. M., Tew K. D., Tapiero H. (2003). The importance of glutathione in human disease. *Biomedicine and Pharmacotherapy*.

[36] Santos-Silva M. C., Freitas M. S. D., Assreuy J. (2006). Involvement of NF-*κ*B and glutathione in cytotoxic effects of nitric oxide and taxol on human leukemia cells. *Leukemia Research*.

[37] Zhang S., Ong C.-N., Shen H.-M. (2004). Critical roles of intracellular thiols and calcium in parthenolide-induced apoptosis in human colorectal cancer cells. *Cancer Letters*.

[38] Orr J. G., Leel V., Cameron G. A. (2004). Mechanism of action of the antifibrogenic compound gliotoxin in rat liver cells. *Hepatology*.

